# The Mystery of Remote Communality: University Students’ and Teachers’ Perceptions During the COVID-19 Pandemic

**DOI:** 10.1007/s42087-021-00262-7

**Published:** 2021-11-25

**Authors:** Satu Uusiautti, Sanna Hyvärinen, Sina Björkman

**Affiliations:** University of Lapland, Rovaniemi, Finland

**Keywords:** Communality, Sense of communality, University community, COVID-19, Remote work, Online interaction

## Abstract

The aim of this study was to examine how the coronavirus disease (COVID-19) pandemic affected the communality among university personnel and students. Herein, we report the findings from a northern Finnish university in which student (mean number of participants, *n* = 339) and staff (mean number of participants, *n* = 133) perceptions and experiences were surveyed. The data (gathered between March 2020 and June 2021 in 7 surveys) included responses to questions about satisfaction with remote and hybrid work and communality. In addition, as the focus of the analysis in this research, we collected the participants’ descriptions of their experiences and thoughts through open-ended questions. The sense of communality among the students, students and teachers, and staff differed, but the appreciation for flexible interactions and availability was similar between the groups. This study discusses the importance of retaining a sense of communality during and after abnormal times on the basis of the reported findings.

## Introduction

We have faced a global pandemic due to the outbreak of coronavirus disease (COVID-19), and the chain of events exempted no one. As the pandemic spread and persisted with new waves, research data about its effects, not only its health and economic effects but also its widespread social effects, had accumulated (González-Zamar et al., 2021; Kniffin et al., 2021; Odriozola-González et al., 2020; Shaw et al., 2020). According to Waters et al. (2021b), the global pandemic is a collective phenomenon. Most workplaces such as educational institutions started following their members’ strategies to cope with the situation and to support their staff and students during the time when their well-being was threatened (Van Agteren et al., 2020). In this paper, we report our findings from our investigation of these pandemic-related activities.

This research focused on higher education students in Finland. During the pandemic, almost a third of university students had severe burnout (STT, 2020), whereas, in previous years, the rate of burnout was only approximately 7% (Salmela-Aro & Read, 2017). According to the Finnish Education Evaluation Center (2021), the shift to online teaching during the lockdown went well, but more attention should be paid to students’ well-being and integration into the higher education community (Repo et al., 2020). The same trend of increased burden was observed among university teaching staff (Tieteentekijät, 2020).

This article reports the findings from the University of Lapland (UoL), a university within the Arctic Circle, in Finland. It is the northernmost university of the European Union, but even with its distant location, the university could not avoid being affected by the pandemic. The UoL is a relatively small higher education institution with a reputation for high communality and vivid interactions between students and teachers. For example, staff share university diners, cafeterias, and corridors with students all the time. This sense of being “easy to approach” and “teachers having time for every student” has been one of the key features reported in earlier surveys to be appreciated by students and particularly representative of the UoL (Student Union of the University of Lapland, 2021).

During the pandemic, the sense of communality, interactions, and face-to-face encounters was suddenly fractured everywhere. This was alarming and concerned people widely at the UoL. In this study, we focused on analyzing the perceptions of communality among university students and staff comparing the changes during the pandemic starting from the first questionnaire sent to students and staff in March 2020 until the end of June 2021. During the study period, the university went from a total lockdown (in March to May 2020), when all teaching and work-related activities were conducted online and the campus was closed, to limited teaching and on-site working with strict hygiene and safety instructions and limitations for groups (ranging from 6 to 50 persons per room from fall 2020 to spring 2021). Finnish universities operated continuously during the pandemic, even when university premises were closed and some regions in Finland were restricted. University staff worked from home, as did the students.

According to the International University Association (International University Association, 2020), universities across the globe reported that they had the infrastructure to communicate with their students and staff during the pandemic (especially during the lockdown). Two-thirds of the universities reported that classroom teaching was replaced by distance teaching, which provided a mandatory opportunity to test and blend various forms of synchronous and asynchronous teaching, learning, and working methods. While the IUA survey revealed an increase in community engagement, the report did not include any notions about perceptions of communality per se. Community engagement in the IUA report referred to activities such as medical advice and support. The purpose of this research was to discuss how the perceptions of communality changed, the lessons learned during the pandemic about the university community, and strategies to ensure that the sense of communality and reciprocal relationships will be maintained in the future.

## Viewpoints on the Meaning of Communality in Extraordinary Situations

The connection between people, that is, the sense of communality, can be viewed from various perspectives. Reciprocal and supportive social relationships are one of the main pillars of human well-being (Seligman, 2011) and development (Berscheid, 2002). Communality between people, on the other hand, is based on communion that “arises from strivings to integrate the self in a larger social unit through caring for others and involves such qualities like focus on others and their well-being, cooperativeness, and emotional expressivity” (Abele & Wojciszke, 2007, p. 751).

Communality, therefore, differs from solidarity or cohesion, being a wider concept that can, however, be explained through the emergence of solidarity and strength of cohesion (Aro, 2011; Kangaspunta, 2011) and the sense of community (Procentese et al., 2019). Furthermore, we are all members of various communities, and the sense of communality can also occur even if the habitual ways of acting together change in the community (Calgano, 2012). The ongoing technological development also has a fundamental impact on communities and communality (Hampton, 2016). However, being a member of a community, whether online or offline, does not automatically lead to a sense of communality (Kuukka et al., 2019; Swan, 2002).

In this article, we use the concept of communality to illustrate the perceptions of members of the community about their togetherness, quality of collaboration and interaction, and sense of community (Clark & Mills, 1993). Communality is thus a positively oriented description of a community that shows communal strength (Mills et al., 2004), which in this case is comprised of university staff and students and manifest not only at the university level but also in smaller groups such as student groups, groups of faculty members, or mixed groups of university staff and students.

According to Abele and Wojciske (2007), secure and friendly relationships are essential for people’s ability to cope because they provide trust, empathy, and support. In the workplace and study contexts, these types of relationships form the basis for functional work and study atmosphere and are mainly established in daily encounters (Karima & Uusiautti, 2018; Määttä & Uusiautti, 2018; Uusiautti, 2016). While employees and students who have been working and studying, respectively, at the university, longer have an established sense of community, newcomers may perceive communality in a different way if face-to-face encounters are limited (Asikainen et al., 2018). Therefore, one aspect of communality during the pandemic is how well the new students and staff members have become members of the community (Prasad et al., 2020).

In their latest research related to the COVID-19 situation, Waters et al. (2021a) distinguished between positive interpersonal processes and high-quality connections (HQC) as significant factors when coping with the pandemic situation or other crises. Positive interpersonal processes refer to everyday experiences that we share with others; for example, we show kindness and love and express our emotions to others (Algoe, 2019; Waters et al., 2021a). Even laughing or joking together as an interpersonal process was interrupted in many ways by the COVOD-19 pandemic. Instead, HQCs refer to interpersonal moments in which people receive positive regards and feel a sense of connection with others (Waters et al., 2021a). HQCs do not necessarily require face-to-face encounters but can transpire also in online meetings and sessions and can be quick or last longer. However, HQCs are important to human well-being and have numerous positive consequences such as better collaboration and resilience (Waters et al., 2021a). Whether in terms of interpersonal processes or the quality of interpersonal connections, COVID-19 changed the nature of the social support in workplaces (Shaw et al., 2020) in the era of increased demand for collaboration and communality at work (Bersin et al., 2017). For example, owing to the lockdown situation, all interactions shifted from the campus to online, including teaching, studying, working, and other formal and informal encounters.

From university students’ perspectives, a study previously conducted by Uusiautti et al. (2017) before the pandemic showed that an ideal online teaching and learning environment include four main components, of which two concerned interaction and relationships. That is, the students emphasized the importance of an active and positive online teacher who cares for the students and the quality of teaching and opportunities for different levels of interaction with peers and the teacher (Keengwe & Kidd, 2010; Rasi & Vuojärvi, 2018). Positive learning experiences are equally crucial in universities as they are at other educational levels (Leskisenoja & Uusiautti, 2017; Rowe et al., 2015). On the other hand, the study by Alves et al. (2021) showed the need for support in teachers’ emotional management and digital skills during the pandemic in Spain.

Several studies have shown that university students and teachers tend to perceive encounters and communality differently (Henderson et al., 2017; Procentese et al., 2019; Prosser & Trigwell, 1997). On the basis of their research, Asikainen et al. (2018) suggested that more attention should be given to the differences in, for example, communality perceptions at the higher educational level. From the perspective of our research, this is a relevant notion and explains the extent of students’ and staff’s perceptions of communality in their mutual and own communities during the pandemic is important.

## Methods

This study was conducted to address the following research questions:

1. How did the perceptions of communality appear among students and staff during the exceptional study and work circumstances during the COVID-19 pandemic?
2. What were the key successes and challenges identified by students and staff regarding communality?

The data used in this research were obtained from surveys designed for university students and staff. The purpose of the surveys was to observe how students and staff coped during the pandemic and to determine the kinds of support they needed as the situation persisted. The survey questionnaires were sent via e-mail to all staff and students simultaneously. The survey questionnaires were compiled using the Webropol online survey tool, where they could be answered on a computer, tablet, or phone. The survey questionnaires were bilingual, with Finnish and English versions available. All the responses were collected anonymously, which meant that it was impossible to know who answered the questionnaire and to combine individual student or staff member responses between the surveys. The students and staff were informed how and why the surveys were conducted and that the findings would be openly shared, albeit in a manner that no individual person could be identified from the reports.

Both the staff and student survey questionnaires contained questions about one’s satisfaction with the work/study arrangements, the support needed and received, the sense of communality, and one’s own motivation to work/study remotely or in hybrid conditions. These structured questions were in the form of statements that the participants had to evaluate as to how well they described them by using a Likert scale ranging from 1 to 5 (1 = completely disagree; 2 = partly disagree; 3 = do not agree or disagree; 4 = partly agree; and 5 = completely agree). In addition, the same open-ended questions about successes and challenges during the period in question in each survey were presented to both the students and staff.

The staff was also asked questions regarding their background information, including sex, age, and staff group, whereas the students marked their sex, age, and department. Owing to the small size of the university, the staff were not asked to mark their departments, as this would probably compromise the respondent's anonymity. The survey questionnaires were sent seven times (Table 1) to the students and staff, always to everyone.

**Table 1:** Survey schedule and participants

Survey time	Number of staff members	Number of students	Special remark
March 2020	210	377	
April 2020	160	333	
May 2020	118	202	
September 2020	111	424	New questions related to the hybrid model of work were included. These questions concerned the safety of work and the progress of contact teaching
October 2020	86	425	Owing to the rapid decrease in the number of participants among the university staff, the monthly surveys were discontinued
January to March 2021	122	393	
April to June 2021	126	219	Two new open-ended questions about how teaching and working should be arranged after the pandemic were included in accordance with the respondents’ suggestion. These questions replaced the questions about successes and challenges

As the pandemic situation changed and prolonged, some modifications were made to the questionnaires. For example, the surveys were launched during the lockdown, but the pandemic continued after the spring semester of 2020. Therefore, the fall survey questionnaires of 2020 were slightly revised to focus on the hybrid model (as the spring questionnaires used the terms “remote work” and “remote studying”) of working and studying because universities were again opened but with some restrictions to, for example, the sizes of the study groups. At the beginning of the year 2021, it seemed unnecessary to continue the monthly surveys, so only two surveys were conducted in the spring of 2021, one focusing on January–March experiences and the other on April–June experiences. In addition, the June 2021 survey included questions about post-COVID-19 teaching and working arrangements, as the pandemic seemed to be nearing its end. Table 1 shows a summary of the surveys and the numbers of survey participants. The response rates were difficult to determine. However, when we compared the number of respondents to the number of staff at the end of the year 2020 (*N* = 614), the overall response rate of the staff ranged from 14% (10/2020) to 34% (3/2020). The number of attending students in 2020 was 3768; therefore, the response rate of the students ranged from 5% (5/2020) to 11% (10/2020). Among the students who participated in all the surveys, 50% were 25 years old or younger. They were mostly women (women 80%; men 19%; and other 1%). Most of the staff were 46 years old or older (53%) and women (women 70%; men 29%; and other 1%).

The data analysis focused on the parts of the surveys that measured communality, collaboration, and satisfaction with work and studies conducted during the pandemic. From the staff surveys, the basic frequencies of the following questions were analyzed in this study: “I feel communality in my hybrid work relations,” “Cooperation forms a challenge in hybrid work,” “Remote teaching went well,” and “Students were happy with the remote teaching arrangements.” From the student surveys, the following questions were analyzed in this study: “Remote teaching was implemented well,” “Remote teaching motivates me,” “I would have needed more support for my remote studying,” and “I feel communality in my hybrid studying community.”

For the numerical data, we were interested mainly in changes about satisfaction and communality and differences in perception between the staff members and the students. Basic frequencies were therefore calculated, keeping in mind that the data included some flaws (e.g., changes in the number of participants and inability to identify respondents in different sets of data). However, the emphasis in this study was on the nature of communality perceptions and not so much on numerical data. Means and medians were used to illustrate the students’ and staff’s evaluations of their satisfaction and communality during the pandemic.

In addition, the answers to open-ended questions were analyzed. The analysis proceeded in accordance with the guidelines for the content analysis method (Mayring, 2014). The analysis was started by reading all open-ended answers and highlighting those that described communality and its preconditions (e.g., mentions about positive interactions or lack of help in remote work). After that, these were divided into categories that represented various communality perceptions (e.g., getting to know each other). Finally, these perceptions were analyzed according to the different group members because communality perceptions were found to be somewhat different among students than among staff. This decision led to the final arrangement of the findings into three groups: communality among students, communality among students and staff, and communality among staff.

## Findings

### Satisfaction and Communality Experiences during Remote and Hybrid Work

#### Student Perceptions

We start by examining student experiences from March 2020 to June 2021 (Table 2). The first two questions (“Remote teaching was implemented well” and “Remote teaching motivates me”) described the overall satisfaction with the teaching arrangement during the time of remote and hybrid work. As the lockdown started in March 2020, satisfaction was somewhat average. The mean satisfaction scores increased until the end of the study year, whereas the medians did not change in 2020. The same trend was found in how motivated students perceived remote teaching. The lockdown was suddenly imposed; thus, once doors were closed, everything had to be transformed into an online environment. Although some courses were already set up, some had to be revised quite comprehensively. Teachers who were not so familiar with the online teaching and learning environments had to learn to use new teaching tools quickly. They also had to inform students about the changing arrangements.

**Table 2:** Student perceptions of study satisfaction and communality in remote and hybrid work (1 = completely disagree; 2 = partly disagree; 3 = do not agree or disagree; 4 = partly agree; 5 = completely agree)

Question	Survey time	Number of participants	Mean	Median
Remote teaching was implemented well	March 2020	377	3.5	4
	April 2020	333	3.6	4
	May 2020	202	3.8	4
	September 2020	424	3.6	4
	October 2020	425	3.5	4
	January–March 2021	393	3.3	3
	April–June 2021	219	3.8	4
Remote teaching motivates me	March 2020	377	2.7	3
	April 2020	333	3.1	3
	May 2020	202	3.1	3
	September 2020	424	2.8	3
	October 2020	425	2.7	3
	January–March 2021	393	2.5	2
	April–June 2021	219	3.4	4
I would have needed more support for my remote studying	March 2020	377	2.5	3
	April 2020	333	2.5	2
	May 2020	202	2.4	2
	September 2020	424	2.7	3
	October 2020	425	2.8	3
	January–March 2021	393	3.1	3
	April–June 2021	219	2.5	2
I feel communality in my hybrid/remote study community	March 2020	377	2.1	2
	April 2020	333	2.4	2
	May 2020	202	2.5	2.5
	September 2020	424	2.2	2
	October 2020	425	2.1	2
	January–March 2021	393	1.9	1
	April–June 2021	219	2.3	2

The events in March 2020 occurred merely as a response to chaos, and April started to appear as a better month in the open-ended answers. Teachers and students became more familiar with the online teaching tools, and the courses began. This interpretation is based on the increase in the number of users in online teaching and learning environments. In May 2020, the study year was about to end, and the teachers and students worked online to complete their degrees, prepare for a summer holiday, and most importantly, they expected things to become normal in the fall.

Thus, when the new study year 2020–2021 started, expectations were high because the students and staff seemed to be anxious to go back to normal. This was constantly stated in the open-ended answers to the survey. The university was opened, but only small-group teaching could be arranged. The use of premises was restricted and meant only for students’ independent use (e.g., computer classes or art classes), use of the library, and university premises in general, as the students normally would have a 24/7 access to the premises with their personal keys. As the situation prolonged and the return to normal was not possible, the students’ satisfaction and motivation decreased toward the end of 2020. However, again in the spring, the same trend was observed, and by the end of spring semester 2021, regardless of restrictions and remote teaching, both satisfaction and motivation seemed to increase.

These changes in satisfaction with teaching arrangements and motivation to study provide an important outlook on what the students and staff were experiencing. This background also sheds light on the experiences of communality. The question about the needs for support shows that, alongside increases in satisfaction and motivation, the need for more support decreased. This was quite expectable and revealed that as the teachers’ skills and abilities improved, students’ needs were also better noticed. Alongside the teachers’ increasing skills, other types of support (e.g., tutoring and student healthcare) became more accessible online.

Regarding communality, students thought about their peers and their own abilities to build relationships, get to know each other, and form study and friend groups. The means and medians in this matter were low, and the sense of communality was clearly not reached during the remote and hybrid work periods. The mean was the lowest at the beginning of 2021 (Table 2: mean, 1.9 and median, 1). We speculate that the impact of the start of the remote study year, new students not being able to integrate into the university community, and the inability to study together accumulated at this point. Again, when the summer holiday drew closer, the pandemic seemed to have slowed down, vaccinations began, and the student responses were also more positive in general.

#### Staff Perceptions

Regarding staff experiences, we introduce findings about satisfaction with online teaching and communality and cooperation (Table 3). The university staff was asked about their satisfaction with remote teaching (those who had been teaching answered this question). In addition, they were asked to evaluate how happy students were with the teaching arrangements. This was to reveal how well the staff observed student reactions and whether their own satisfaction was in line with the evaluated satisfaction of students.

**Table 3:** Staff perceptions of satisfaction and communality during remote and hybrid work (1 = completely disagree; 2 = partly disagree; 3 = do not agree or disagree; 4 = partly agree; 5 = completely agree)

Question	Survey time	Number of participants	Mean	Median
Remote teaching went well	Mach 2020	210	3.5	4
	April 2020	160	3.7	4
	May 2020	118	3.9	4
	September 2020	111	3.7	4
	October 2020	86	3.8	4
	January–March 2021	122	3.8	4
	April–June 2021	126	3.5	4
Students were happy with the remote teaching arrangements	March 2020	210	3.6	4
	April 2020	160	3.8	4
	May 2020	118	4.1	4
	September 2020	111	4.0	4
	October 2020	86	3.6	4
	January–March 2021	122	3.6	4
	April–June 2021	126	3.5	4
I feel communality in my hybrid work relations	March 2020	210	3.6	4
	April 2020	160	3.6	4
	May 2020	118	3.3	4
	September 2020	111	3.6	4
	October 2020	86	3.5	4
	January–March 2021	122	3.1	3
	April–June 2021	126	3.5	4
Cooperation forms a challenge in hybrid work	March 2020	210	2.8	3
	April 2020	160	2.7	2
	May 2020	118	2.9	3
	September 2020	111	2.9	3
	October 2020	86	2.9	3
	January–March 2021	122	3.0	3
	April–June 2021	126	2.7	2

In general, the staff’s responses were more positive than those of the students. However, the same trend as that observed in the students' perceptions was found as the pandemic prolonged. While the mean values of the satisfaction and perception of the students’ happiness increased by the end of study period 2019–2020, the decrease became visible when the first semester of study period 2020–2021 ended. The decrease continued until the end of spring 2021. However, the medians did not change. Interpretations could be made, however, on the basis of the open-ended answers that will be introduced in the next section.

The staff was also asked about challenges with cooperation in their work communities. All members of the staff answered, including those with no teaching responsibilities. The mean values remained somewhat the same during the whole study period and peaked in March 2021. However, this change was not observed in the median values, but the best times for collaboration were in April 2020 and the end of spring 2021.

The staff’s sense of communality, while being good on average, was the lowest also when the students’ corresponding measurement was lowest in March 2021. The median value for sense of communality was only 3 in this survey. When reexamining the societal discourse at that time, we found that mental health issues, stress, and burden emerged not only among students but also among the staff. Indeed, this time appeared to be the culmination point of our survey and the general national discussion of these matters at higher education in Finland.

### Communality Perceptions in Open-Ended Questions

#### Communality Among Students

Several factors seemed to hinder the development of communality among students. Getting started with the studies was considered challenging. Misunderstandings could arise in getting acquainted with groups and university studies while working online. Students would have preferred to be in the company of peers and teachers. The students also wished for more support from their peers, as expressed in the following statements:

“A new study group of complete strangers and you cannot meet them. Working alone on a new topic and studying at a university for the first time, so the changing schedules and arrangements created some confusion.”

“Not being able to be on campus to attend lectures and to see people. The hardest thing for me is that you cannot be so much in real contact with people, which is why it felt weird to start the autumn term.”

The versatile methods and flexibility of the courses were regarded as the most important streaks of success. Working in small groups during online teaching stood out as a well-functioning practice among the student replies about their communality experiences. Division into small groups went well technically, and the teachers often supported group work. The students also felt that working in small groups increased group identification and the sense of belonging, as expressed in the following responses:

“The technology has worked well, and the teachers try to keep us updated on changes. The group itself has unified a bit, and the members have asked for help from one another, for instance, on Facebook.”

“I think that small-group work has advanced group identification now at the beginning of our studies.”

“The fact that when the camera is on, interaction increased at least in the small group… An inspiring teacher also inspires the students… The teacher made regular visits to each small group space. I think it worked well.”

By and large, providing students with a chance to catch up and arrange time and space for interactions was considered positive study experiences.

“I think there’s been a sense of community on my thesis course, as we have discussed how everyone is doing. It’s felt so good to talk with the course companions. I just love the teacher, so nobody has been uptight at all. I feel that it starts from the teacher, communality, and then students can join in and share things easier.”

The students suggested paying attention to successful interactions and their active promotion during the lectures. They also suggested better possibilities for small-group work or discussions on mass lectures and for discussion sessions before or after lectures on an online work platform. This is partly because, otherwise, online lectures would not have any discussion culture, which takes shape during breaks or after lectures in normal contact teaching.

“Teachers must encourage students to use the microphone and talk/participate in discussions.”

“There is no discussion culture during lectures.”

Teachers could help students build their mutual sense of communality via various online teaching arrangements. In addition, the students interacted flexibly with each other. The student unions arranged virtual events and peer support. Still, many students reported exhaustion in studying alone and misery due to the lack of interactions in their student life. Loneliness was often mentioned in the students’ answers.

#### Communality Among the Students and the Staff

As the students described the challenges of scheduling, motivation, and self-discipline, they wished for more personal support from their teachers. Many thanked their teachers for their detailed and timely communication, although some obscurities remained. The students reported that among their most important experiences of success during the COVID-19 pandemic were the completion of their courses and the practices in remote studying that contributed to the quality of their studies, as depicted in the following:

“Communication and a willingness to help and availability (e.g., quick e-mail replies), regardless of the difficult situation.”

“We have a course where the teacher is made of pure gold. He/she communicated excellently about the effects of the COVID-19 situation and kept us up-to-date. We always knew what was required from us, and the schedules were clear. Normally, it would not even be possible to carry out this course online... but this teacher did a good job in spite of the circumstances.”

“I noticed I was working more than before and preparing in advance for lectures. This also made it easier for me to participate in discussions. By doing so, I also learned more from the courses.”

The experiences of success emphasized by the staff dealt with the organization of work, introduction of new tools, students’ flexibility, success of meetings, and personal learning. The teachers appreciated positive feedback and encounters with students. The students’ enthusiasm was inspiring and motivating.

“Students reacted well to the changed situation—everyone embraced it with enthusiasm and adapted quickly to the new practices.”

“The technical success of online lectures and students’ active participation in online teaching.”

“Positive feedback from students.”

In online and hybrid teaching, the arrangements had to be revised, and the teachers had to be prepared to come up with flexible solutions. The teaching staff was grateful to the students, whose activeness and enthusiasm were mentioned in many replies to the open-ended questions.

“Motivated students and their almost 100% attendance.”

“Students’ active participation in an online workshop. They didn’t seem to mind working online, as their participation was really active regardless.”

However, mutual understanding between students and teachers was highlighted in both respondent groups. This means that the teachers showed understanding about the students’ difficulties in studying alone or being isolated from others and their various situations. On the other hand, gratitude was expressed for effort and hard work from the students to their teachers. These positive experiences were mentioned in the open-ended answers.

“Motivation from professors/staff not only for studying and coping but also for resting has been important. The staff’s awareness of the fact that the situation is not easy for students and that the pace is not the same as usual has been a relief.”

“Positive feedback from international postgraduates on online meetings and supervision.”

The students were brought up having contact with teachers and the possibility for either individual or group guidance and discussions. As for the teachers, the students wished for informal small-group meetings supervised by a teacher, online appointments, accessibility and courage to use various communication devices, and individual online guidance.

“Teachers have provided personal guidance through AC [Adobe Connect]; this is really effective and educational.”

“I wish teachers would tip one another off.”

The third issue pointed out by the students was the importance of using a camera during online studying. Of particular importance was seeing the teacher live, and student cameras were considered a plus. In terms of communality and interaction in general, students also thought of an online café that one could visit remotely. Students also wished for more joint online events.

#### Communality Among the Staff

In answer to the open-ended questions, the respondents described that they had learned new practices and were enthusiastic but brought up pressures and the strenuousness of remote work. As to their perceptions about communality, the staff was happy with unofficial encounters, virtual coffee breaks, and chats. For some respondents, the increased number of online meetings also increased their experienced communality.

However, communication challenges remote work, and cooperation was developed clearly. Many respondents considered the activation of quiet participants and enabling their participation in meetings challenging. On the other hand, interaction in online meetings was often imbalanced owing to the various skill levels and activities of colleagues. Informal encounters were longed for.

“The lack of community, casual conversations that often ‘accidentally’ inspire you to come up with new ideas, loneliness.”

“Connection to my team is next to zero and basically depends on e-mail… My boss seems to lack any skill in leading the team in remote and hybrid work.”

“In principle, things are okay, but I’m totally exhausted with this remote mode, and I really miss communality and human contact. I’m about to lose my fighting spirit, as I was able to fight last year, and the spirit has gone down a lot recently.”

According to staff, the best way to promote communality and interaction was to arrange virtual coffee breaks for handling work-related issues and facilitating informal discussions. WhatsApp groups, messaging, and “how are you” meetings function as communication channels and ways to keep updated with the members of small and larger groups. Moreover, larger meetings such as Rector’s virtual coffee breaks for the whole community were welcomed, and people hoped to see more of them in the future.

“For example, ‘Teams coffee breaks’ make me feel that I am still part of the group.”

“In my experience, online meetings are as productive as face-to-face ones. In fact, they can be even more efficient than face-to-face meetings.”

Walking meetings were also suggested if the topic allowed one to do so. Ideas on joint exercise breaks were also presented. The replies encouraged people to be open-minded and innovative in making their remote work environment interesting and comfortable.

## Discussion

Our findings show that the need for communality emerged among both the students and staff. The data also included those who enjoyed working online and thrived in solitude. For future work, it is crucial to develop flexible working and studying conditions. However, for the sense of communality, face-to-face encounters at the campus were generally missed. Although the staff’s and students’ skills with remote work increased considerably during the period under analysis, a common experience seemed to be that the lack of non-verbal communication hindered interactions and seemingly decreased the sense of communality (Sekerdej et al., 2018; Simão & Seibt, 2015).

In addition, the differences in satisfaction level and sense of communality between the staff and the students show that many factors influenced the experienced stress and resilience during the abnormal situation (Odriozola-González et al., 2020; Van Der Feltz-Cornelis et al., 2020). This suggests the extent of the impact of the COVID-19 pandemic and that support measures should be developed to address the varied experiences resulting from the situation. The sense of communality can be understood as an aspect that needs more attention in universities during and after the COVID-19 pandemic.

On the other hand, the staff and students had invented creative ways of encountering (e.g., meetings via phone while walking, online coffee breaks, and online student events). Osler’s (2020) research in this respect is worth mentioning. She studied communality in online encounters by investigating habitual communal experiences and actual we-experiences. Her research suggested that online experiences resembled face-to-face encounters to some extent and that communality can be attained when people meet online.

The new ways of working and meeting online seemed to retain communality, at least to some extent. Calgano (2012) uses the concepts of communal retention or memory. On the basis of this research, it seemed crucial that staff retains communal memory. This was observed in at least two scenarios: in the need for finding new ways to encounter and communicate (officially and unofficially) and as the fear of losing connection or sadness for the lost connection with the work community. Regarding the communality between teachers and students, successful online teaching arrangements that enhanced interaction and reciprocal positive feedback and availability appeared as the key measures to cope with the situation (Wood et al., 2010). The situation among students was complex, as new students found it difficult to integrate into the student community, and the traditional student life was mostly missing. Some students could stay in their homes and have their families as social support, whereas some were staying in Rovaniemi, alone in student apartments, and thus longed for peer support.

### Limitations

Certain limitations must be considered when evaluating our findings. First, the study focused on just one university; therefore, the results cannot be generalized to other universities. Nevertheless, the experiences highlighted in the data provide a deeper understanding about how students and staff perceived the pandemic situation and its impacts in their study and work conditions, respectively. The strength of the study is that the respondents described their perceptions and experiences openly, showing both successes and difficulties and thus providing a multidimensional and credible picture of the phenomenon.

Another limitation was that communality was assessed using a simple measure in the structured questions of the survey. Therefore, only the frequencies of the questions related to communality during remote and hybrid work could be presented. Clearly, if the original survey was aimed at measuring only the perceived communality, more structured questions about it could have been included. As the survey also considered other aspects of remote and hybrid work, in this study, only those focusing on communality were included in the analysis.

However, as mentioned earlier, the emphasis in this research was in qualitative analysis, that is, in the data obtained from the open-ended questions. Together, our findings provide a glance at the communality experiences of students and staff. The means and medians in the quantitative data provided the initial findings about the general perceptions, whereas the qualitative analysis of the participants’ descriptions in their own words provided a more in-depth analysis of meaningful experiences (both good and bad) during the pandemic.

Furthermore, this study is limited owing to the relatively low response rates of the students, at least when compared with the response rates of the staff. However, given the burdens conferred by the pandemic, the number of respondents was considered satisfactory. In addition, the initial purpose of the survey was to listen to the members of the community; in this sense, the survey was effective, as the open-ended answers were detailed and honest. The community was provided with a summary of the results after each survey, which was hoped to encourage the students and staff to participate in the survey.

As the core of the survey remained the same the whole time, the respondents were at risk of satisficing (Vannette & Krosnick, 2013). To avoid this, slight changes were made to the open-ended questions and survey loops. However, the staff and students seemed to find it meaningful to answer the questionnaire and express their thoughts, share their experiences, and to wish for improvements and future arrangements.

## Conclusion

This study examined the communality experiences of students and staff in a Finnish university during the COVID-19 pandemic. The importance of understanding how the sense of communality is retained was evident. Some new and excellent ways of working and studying together were discovered, but some worrying aspects of full online or remote work were identified. Future studies should clarify what will happen next and whether the sense of communality can be revived or changed somehow.

Waters et al. (2021b) discussed the posttraumatic growth of communality. Indeed, not only observing communal well-being after the pandemic (Van Agteren et al., 2020) but also actively supporting positive transformations as those mentioned by Masten and Obradovic (2008) when referring to the increased resilience after disaster will be important. The key support systems in all positive developments are social interactions and attachment (Uusiautti et al., 2022). Therefore, one of the main tasks will be to exert effort on bringing back the sense of communality on campuses, as this will be the building block to many other positive developments after the pandemic.

The main notions and recommendations from this study are as follows:

1. Various types of informal online meetings should be arranged systematically. As this form of interaction is missing, supervisors (for workgroups and teams) and teachers (for their courses and lessons) should plan these types of meetings. For example, online coffee breaks and discussions without agenda were identified as good practices in the survey.
2. Students’ peer relationships in the online community should be paid extra attention. Student unions and organizations play key roles in organizing and arranging informal events and meetings among students. Special attention should be focused on students who are not active members of student communities and providing ways in which students can get to know others and find peer support.
3. Interactions in formal encounters should be enhanced by using cameras and microphones actively and engaging participants widely. During lessons, teachers’ abilities to use small-group tasks, breakouts, and online discussions appeared meaningful to students. The same notion was found in workgroups that met online. Vivid interaction was appreciated by most.
4. Finally, people adjust to changing situations differently. Some students and staff enjoyed the opportunity to work alone or remotely. The other extreme exists as well: other students suffer tremendously from mandatory remote work and studying. The reasons are many, as some do not have a quiet place at home to work while others lack self-motivating skills and need the community around them to accomplish their tasks. Understanding various experiences is crucial; therefore, support for these various needs should be actively arranged during extraordinary times.
